# Rapid detection of FAdV-4 by one-tube RPA-CRISPR/Cas12a assay

**DOI:** 10.3389/fmicb.2025.1541943

**Published:** 2025-02-03

**Authors:** Lei Ma, Xueping Wang, Mingliang Zhang, Mengjie Zhu

**Affiliations:** ^1^School of Biotechnology and Food Engineering, Anyang Institute of Technology, Anyang, China; ^2^College of Animal Science and Technology, Tarim University, AIar, China

**Keywords:** avian adenovirus type 4, Cas12a, recombinase polymerase amplification, detection, sensitivity

## Abstract

**Introduction:**

Fowl adenovirus serotype 4 (FAdV-4) is a highly contagious viral pathogen of global significance that affects various avian species. It primarily infects poultry and wild birds, leading to avian inclusion body hepatitis (IBH) and hepatitis-hydropericardium syndrome (HHS). The development of rapid diagnostic tools for detecting FAdV-4 is crucial for effective disease control and eradication efforts.

**Methods:**

In this study, we developed a recombinase polymerase amplification (RPA) combined with CRISPR/Cas12a assay, specifically targeting the FAdV-4 Hexon gene. RPA and CRISPR/Cas12a reagents were added to the bottom and lid of the test tube at once, allowing the detection process to occur within a single reaction tube. This approach reduced contamination.

**Results:**

The RPA-CRISPR/Cas12a detection method can identify as few as 10 copies of the genome per reaction, demonstrating 100% sensitivity comparable to that of fluorescence PCR (qPCR). This approach exhibits high specificity for FAdV-4, with no cross-reactivity observed with other FAdV serotypes or common avian pathogens. Additionally, the agreement rate between the results of RPA-CRISPR/Cas12a and qPCR for detecting clinical samples is as high as 97.5%.

**Discussion:**

Therefore, the RPA-CRISPR/Cas12a assay presents a promising alternative for the simple, sensitive, and specific identification of FAdV-4.

## Introduction

FAdV, or fowl adenovirus, belongs to the Adenoviridae family and the avian adenovirus genus (Schachner et al., 2018; Wang and Zhao, 2019). Through cross-neutralization and restriction enzyme assays, avian adenoviruses have been classified into five species: A, B, C, D, and E, comprising a total of 12 serotypes (Hess, 2000). Notably, FAdV-1-induced adenoviral gizzard erosion (AGE) is marked by macroscopic lesions in the affected gizzards, including inflammation and ulceration; FAdV-4 is associated with pericardial effusion hepatitis syndrome (HHS); and FAdV-8 causes inclusion body hepatitis (IBH) in chickens (Mo, 2021).

The disease primarily spreads horizontally through the fecal-oral route, although some studies suggest that vertical transmission may also occur. In China, HHS cases were sporadic before 2015; however, since July 2015, the disease has surged dramatically, with reports emerging from multiple provinces, including Chongqing, Hebei, Guangdong, Shandong, Jiangsu, Hubei, Liaoning, Sichuan, and Zhejiang (Wang et al., 2019; Jiang et al., 2019; Yuming et al., 2020; Li et al., 2017; Chen et al., 2019; Yu et al., 2019). In China, FAdV-4 has emerged as the serotype with the highest infection rate among adenoviruses.

Typical features of HHS include the accumulation of transparent or pale yellow fluid in the pericardium, liver swelling with fragility and discoloration, as well as focal necrosis and petechial hemorrhage (Asthana et al., 2013). Histopathological examination reveals characteristic changes in the liver, including small multifocal necrosis and alkaline inclusions in liver cells. Additionally, renal tubular epithelial cell necrosis, infiltration of mononuclear cells in the alveolar wall, and necrosis with inflammatory cell infiltration in the spleen are observed (Asthana et al., 2013; Sun et al., 2019).

Chickens between the ages of 3 to 6 weeks are especially vulnerable to FAdV-4 infection, with mortality rates ranging from 30% to 80% (Kumar et al., 1997). Besides, FAdV-4 infection can lead to a significant reduction in weight gain, as well as negative impacts on various production parameters, resulting in substantial economic losses (Grafl et al., 2012).

The molecular biology detection methods for adenoviruses include polymerase chain reaction (PCR), real-time quantitative PCR, restriction fragment length polymorphism polymerase chain reaction (PCR-RFLP), and loop-mediated isothermal amplification (LAMP) (Wang et al., 2017a; Zhai et al., 2019). While the sensitivity and specificity of molecular biology detection methods have significantly improved, traditional detection methods do have some drawbacks. They often require expensive equipment and skilled operators, and samples may need special processing, increasing operational complexity and time costs.

CRISPR/Cas is a bacterial adaptive immune system that has gained significant attraction in recent years due to its rapid detection capabilities (Wang and Doudna, 2023). The CRISPR-associated protein (Cas) functions by cleaving foreign nucleic acids, guided by programmable CRISPR RNA (CrRNA) that boasts high nuclease activity. Key Cas subtypes include Cas9, Cas12, Cas13, and Cas14. CRISPR/Cas12a (also known as CRISPR/Cpf1) belongs to the second family of Cas enzymes. It recognizes specific protospacer-adjacent motifs (PAMs) that are rich in thymine (T) nucleotides and catalyzes the maturation of its CrRNA. Cas12a specifically targets and cleaves complementary double-stranded DNA while demonstrating robust non-specific trans-cleavage activity toward single-stranded DNA (ssDNA), which generates fluorescent signals. The target double-stranded DNA can be obtained through recombinase polymerase amplification (RPA). RPA is an isothermal amplification method that is quick, cost-effective, and does not require specialized equipment (Kellner et al., 2019). As a result, RPA-CRISPR/Cas12a has become widely utilized for virus, bacteria and parasite detection (Qian et al., 2021; Lin et al., 2023; Xiong et al., 2022; Liu et al., 2021; Zhang et al., 2021).

The purpose of this study is to develop a novel diagnostic method that combines RPA and CRISPR/Cas12a technology, providing a fast and accurate approach for early diagnosis of FAdV-4.

## Materials and methods

### Viruses

Avian influenza virus (AIV), Newcastle disease virus (NDV), Infectious bursal disease virus (IBDV), Marek's disease virus (MDV) and Infectious laryngotracheitis virus (ILTV) were preserved in our laboratories. The details of these viruses have been detailed in one of our previous studies (Talwar et al., 2021). FAdV-1, FAdV-2, FAdV-3, FAdV-4, FAdV-5, FAdV-6, FAdV-7, FAdV-8a, FAdV-8b, FAdV-9, FAdV-10, and FAdV-11 were purchased from China Institute of Veterinary Drug Control (Beijing, China).

### Preparation of DNA molecular standards

To facilitate primer design, we obtained the Hexon gene sequences from various FAdV-4 strains through the NCBI database: isolate YN2109 (OQ605505.1), PDS (KU647685.1), YN1605 (OQ605508.1), WS (KU647686.1), CG-D (KU647683.1), XZ (KU647687.1), ZK(KU647689.1), YZ (KU647688.1). Using DNAMAN v5.0 software, these sequences were compared, and the conserved region of the Hexon gene was cloned by the following method.

Nucleic acids from the FAdV-4 strain were obtained utilizing a DNA/RNA extraction kit from Tiangen (Beijing, China). To amplify the conserved Hexon gene via PCR, we designed a specific pair of primers: the forward primer (5′-GCCCACCCGAAATGTCACGA-3′) and the reverse primer (5′-CCGGTCGGGCAGCTCGACCA-3′). The forward primer and reverse primer are located at positions 243-262bp and 1028-1047bp of the Hexon gene, respectively, with a target gene length of 805bp. The PCR was conducted following the protocols outlined in the PCR Kit Manual (Takara, Beijing, China). The PCR procedure involved an initial denaturation at 95°C for 3 min, followed by denaturation at 95°C for 30 s, annealing at 55°C for 1 min, and extension at 72°C for 30 s, with a total of 30 cycles run; finally, a final extension step was conducted at 72°C for 5 min.

Following the PCR, the products were subjected to agarose gel electrophoresis for analysis and subsequently purified. The resulting purified gene fragment was then cloned into the pMD18-T vector according to the product manual (Takara, Beijing, China). The pMD-18T vector is a commonly used linear cloning vector characterized by the presence of multiple T (adenine) termini at both ends. During PCR amplification of DNA fragments, Taq DNA polymerase generates a poly-A tail at the 3′ end of the PCR product. In the ligation reaction, T4 DNA ligase is employed to join the poly-A terminated DNA fragment to the T termini of the pMD-18T vector. Once the ligation is complete, the resulting recombinant vector was transformed into competent cells DH5α. The authenticity of the recombinant plasmid was verified through nucleotide sequencing. The plasmid successfully inserted with the Hexon gene was named pMD-Hexon. Plasmid extraction was carried out using a reagent kit, and the concentration was determined using a Nano2000 (Thermo Scientific, MA, USA). The recombinant plasmids were utilized as DNA molecular standards.

### RPA primer design

The design principles for RPA primers dictate a minimum length of 30 nucleotides, with typical primers ranging from 28 to 32 nucleotides. It is crucial to avoid excessive palindromic structures; the optimal range of G and C content is between 40% and 60%. Additionally, multiple G residues at the 5′ end of the primer should be minimized, while the 3′ end should ideally contain a higher proportion of G and C nucleotides. The target sequence for amplification typically ranges from 100 to 200 bp.

Based on the aforementioned guidelines, the amplification efficiency of the various primer pairs was assessed via agarose gel electrophoresis, allowing us to select the primer pair with optimal amplification efficiency for use in the CRISPR system. All primers and reporter molecules utilized in this study were synthesized by Sangon Biotech (Shanghai) Co., Ltd (Shanghai, China).

### CrRNA design

CrRNA plays a crucial role in guiding the Cas12a protein recognition of the protospacer adjacent motif (PAM) which is characterized as TTTN (where N denotes any nucleotide). To design CrRNA, a specific sequence is selected followed by the PAM sequence. The CrRNA sequence includes a Hexon gene sequence (about 20 bp) and a scaffold sequence. In addition, a T7 promoter sequence is also added to the 5′ end of the CrRNA sequence. Three CrRNAs were designed. The sequences were subjected to BLAST analysis on the NCBI platform. The results indicated that the sequences exclusively aligned with the gene of FAdV-4, revealing no overlapping sequences in the genomes of other species or among other viruses.

The CrRNA sequence was synthesized and inserted into the pGEX-T vector, which was provided by Sangon (Shanghai, China). The plasmid containing the CrRNA sequence was *in vitro* transcribed by Cleascrip^TM^ T7 RNA Polymerase according to the Kit manual (Yeasen Biotechnology, Shanghai, China). The obtained RNA was used for the subsequent experiments.

### CrRNA screen

To assess the activity of CrRNAs, we performed a CRISPR/Cas12a detection analysis. The assay formulation included 0.5 μL of Cas12a protein (New England Biolab, MA, USA), 1 μL of CrRNA (2 μM), 2 μL of 10X NEBuffer 2.0 (New England Biolab, USA), 0.5 μL of a single-stranded DNA (ssDNA) probe (5′-FAM-TTATT-BHQ1-3′) as a reporter, and 2 μL of Hexon gene obtained from PCR. The reaction tube was placed under the Deao instrument (Guangzhou, China) to detect the fluorescence signal at a temperature of 39°C for 20 min. Fluorescence amplitude was recorded and utilized to evaluate the cleavage efficiency of the CrRNAs. Distilled deionized water (ddH_2_O) served as the negative control.

### One-tube RPA-CRISPR/Cas12a assay

The one-tube RPA-CRISPR/Cas12a assay integrates a RPA reaction with Cas12a detection. The RPA reaction was performed using the RPA Basic kit (TwistDx, Maidenhead, United Kingdom), following the manufacturer's instructions. Each 50 μL RPA reaction comprised a mix of 2 μL of each RPA primer (10 μM), 29.5 μL of buffer, 1 μL of template (DNA molecular standard pMD-Hexon prepared above), and 2 μL of MgOAc (280 mM), all added to the bottom of a tube containing enzyme pellets.

For the Cas12a detection, the reagents-1 μL of Cas12a (10 μM), 1 μL of CrRNA (10 μM), 0.25 μL RNA inhibitor, 1 μL of DNA FQ reporter, and 2 μL of 10 × NEBuffer were placed on the inner surface of the tube lid. Throughout the entire process, the reagents were kept on ice. The tubes were carefully covered and incubated in a metal bath at 39°C for 20 min. Subsequently, the tubes were briefly centrifuged and transferred to a Deao incubator (Guangzhou, China) at 39°C for an additional 20 min. Fluorescent signals were captured at 30-s intervals. Generally, a sample is considered positive if it produces a clear amplification curve and the fluorescence value exceeds the threshold of the negative control; otherwise, the sample is deemed negative.

**Figure 1:**
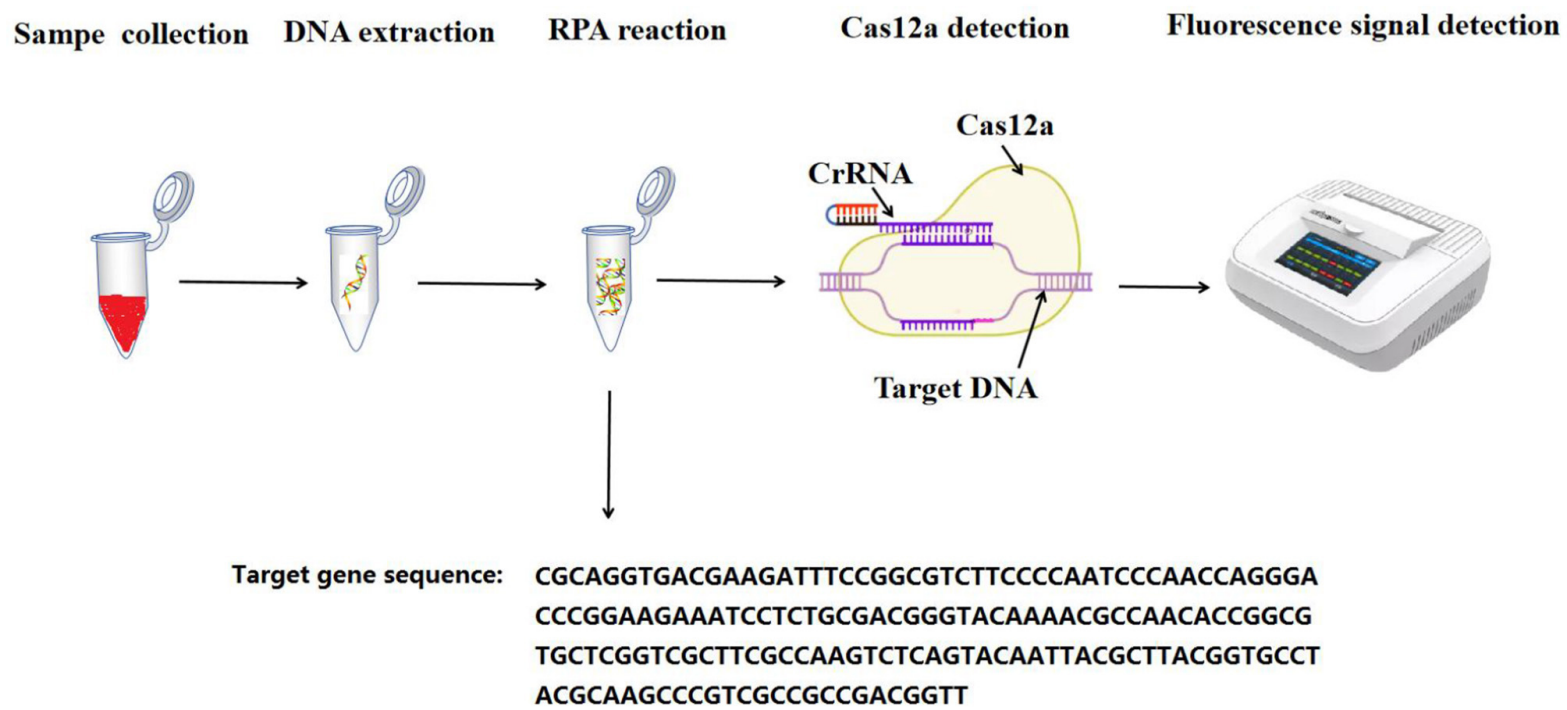
The flowchart for the RPA-CRISPR/Cas12a assay.

The experimental procedure is described in Figure 1.

### Optimization of reaction conditions

In the one-tube RPA-CRISPR/Cas12a detection method, the specific concentrations of Cas12a protein and CrRNA are critically important for achieving optimal detection results. Firstly, the one-tube RPA-CRISPR/Cas12a assay was conducted using various CrRNA concentrations (1.5 μM, 1 μM, 0.5 μM, and 0.25 μM). The optimal CrRNA concentration was identified by analyzing the fluorescence values. Then, the one-tube RPA-CRISPR/Cas12a assay was conducted using the optimal CrRNA concentration, and different Cas12a concentrations (1.5 μM, 1 μM, 0.5 μM, and 0.25 μM) were evaluated as above.

### Sensitivity test

The sensitivity of the one-tube RPA-CRISPR/Cas12a method was evaluated under the optimized conditions established above. The real-time one-tube RPA-CRISPR/Cas12a tests were carried out using the recombinant plasmid pMD-Hexon, which was diluted from 10^6^ to 10^10^ copies/μL to serve as the template. Distilled deionized water (ddH_2_O) served as the negative control. The experiment was conducted in triplicate. In addition, the one-tube RPA-CRISPR/Cas12a method was compared with both the qPCR detection method and the basic RPA detection method. The qPCR detection method has been previously described (Wang et al., 2017a), while the details of the basic RPA detection method are outlined above.

### Repetitive testing

Three DNA molecular standards with varying concentrations (1 × 10^6^, 1 × 10^4^, and 1 × 10^2^) were used as templates. For each plasmid concentration, three replicates were conducted for intra-group repeatability. Simultaneously, each concentration underwent three independent tests for inter-group repeatability. The standard deviation (SD) and coefficient of variation (CV) for each dilution sample were calculated within and between groups to evaluate the method's reproducibility.

### Specificity test

To verify the specificity of the one-tube RPA-CRISPR/Cas12a method for the detection of FAdV-4, the assay was used to detect various avian infectious pathogens. These pathogens are as follows: AIV, NDV, ILTV, MDV, IBDV, FAdV-1, FAdV-2, FAdV-3, FAdV-5, FAdV-6, FAdV-7, FAdV-8a, FAdV-8b, FAdV-9, FAdV-10, FAdV-11. FAdV-4 was included as the positive control, and ddH_2_O was used as the negative control. The experiment was conducted in triplicate.

### Evaluation of the effect of clinical sample testing

From 2020 to 2024, a total of 40 clinical samples were collected from chickens suspected of FAdV infection in a chicken breeder in Yangling, Shanxi province, China. FAdV-4 can lead to symptoms such as hepatitis and pericardial effusion, prompting this study to collect tissue samples from the heart and liver for detection. One heart sample and one liver sample were collected from each chicken, resulting in a total of 40 samples from 20 chickens. These samples were collected for laboratory diagnostics and not specifically for our research purposes. Sample collection operation was approved by the institutional animal care and use committee (IACUC) of Anyang Institute of Technology, Anyang, China. After the samples were collected, the internal organs were chopped using scissors, and 1 mL of buffer solution was added for every 1 g of tissue to prepare a homogenate. To enhance the release of virus particles, the samples underwent three freeze-thaw cycles in a refrigerated homogenization environment. The supernatant was then obtained through high-speed centrifugation, followed by DNA extraction using Qiagen AllPrep DNA/RNA Mini Kit (QIAGEN China, Shanghai, China) following the instructions in the product manual. Finally, the extracted DNA was analyzed by the RPA-CRISPR/Cas12a method, and the results were compared with those obtained using the qPCR method.

### Data analysis

GraphPad Prism 8.0 was utilized for data analysis and processing. The Student's t-test was employed to assess significant differences between groups. A *p* < 0.05 indicated significant differences, while a *p* > 0.05 suggested no significant differences. Additionally, a *p* < 0.01 denoted highly significant differences.

## Results

### Sequence alignment

**Figure 2:**
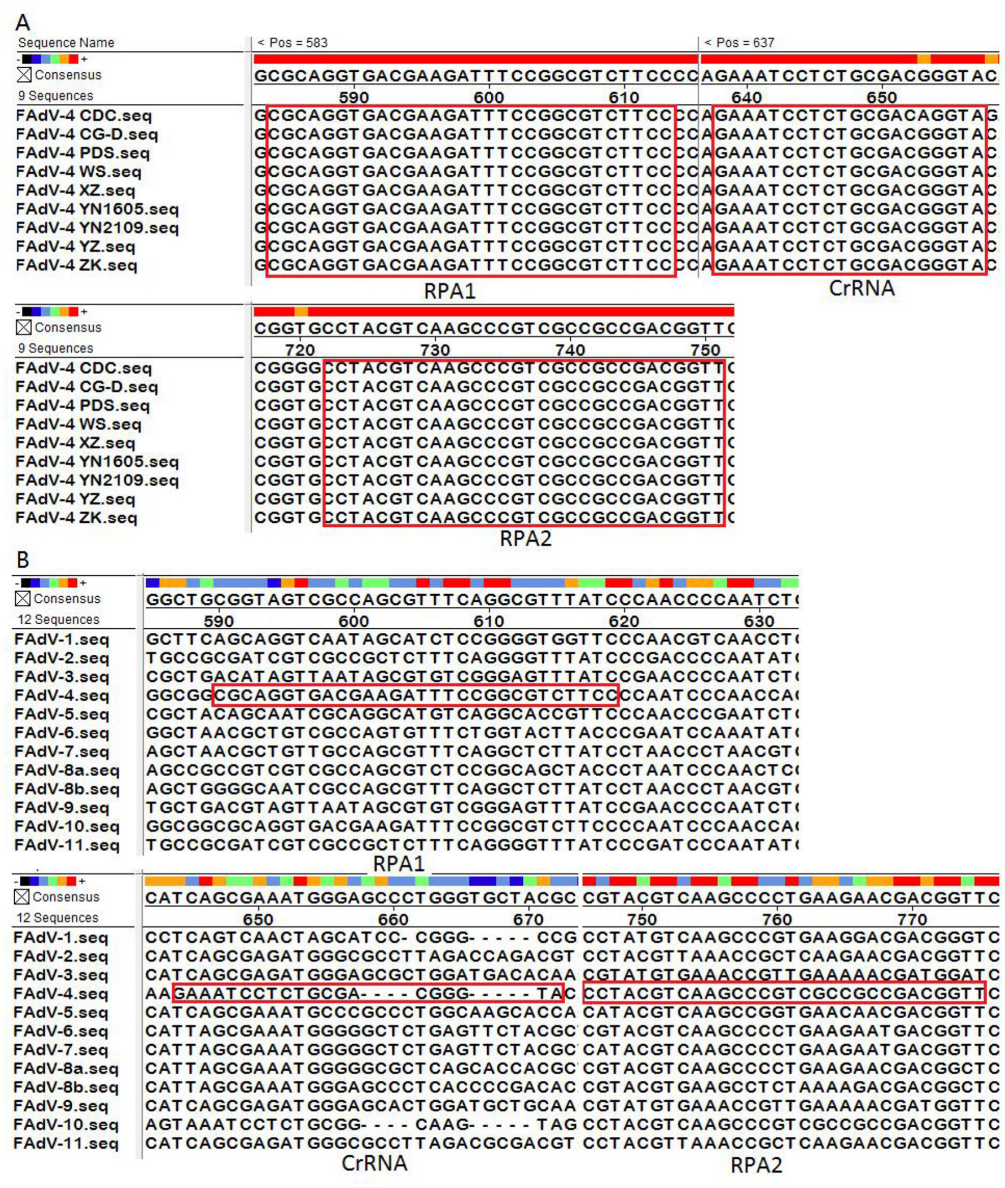
Sequence alignment. **(A)** The analysis of the homology of Hexon genes among different FAdV-4 strains; **(B)** The analysis of the homology of Hexon genes among different FAdV serotypes (FAdV-1 to FAdV-11). The RPA primers (RPA1 and RPA2) and CrRNA were marked in the red box.

The sequences of the RPA primers and CrRNA were shown in Table 1. Because the CrRNA2 performed well in the following section, only the sequence of CrRNA2 was analyzed. The analysis of the homology of Hexon genes among different FAdV-4 strains was displayed in Figure 2A. There is only one base difference among the nine FAdV-4 strains. The analysis of the homology of Hexon genes among different FAdV serotypes (FAdV-1 to FAdV-11) was displayed in Figure 2B. As show in Figure 2B, the sequence differences among different FAdV serotypes are highly significant. The primers and CrRNA sequence of FAdV-10 shows the highest homology with FAdV-4, differing by five bases.

**Table 1:** The sequences of the RPA primers and CrRNAs.

**Name**	**The sequence (5^′^-3^′^)**
RPA1	CGCAGGTGACGAAGATTTCCGGCGTCTTCC
RPA2	AACCGTCGGCGGCGACGGGCTTGACGTAGG
CrRNA1	UAAUUUCUACUAAGUGUAGAUTTCCGGGTCCCTGGTTGGGA
CrRNA2	UAAUUUCUACUAAGUGUAGAUTACCYGTCGCAGAGGATTTC
CrRNA3	UAAUUUCUACUAAGUGUAGAUGTACCCGTCGCAGAGGATTT

Red words: the scaffold sequence.

### CrRNA screen

**Figure 3:**
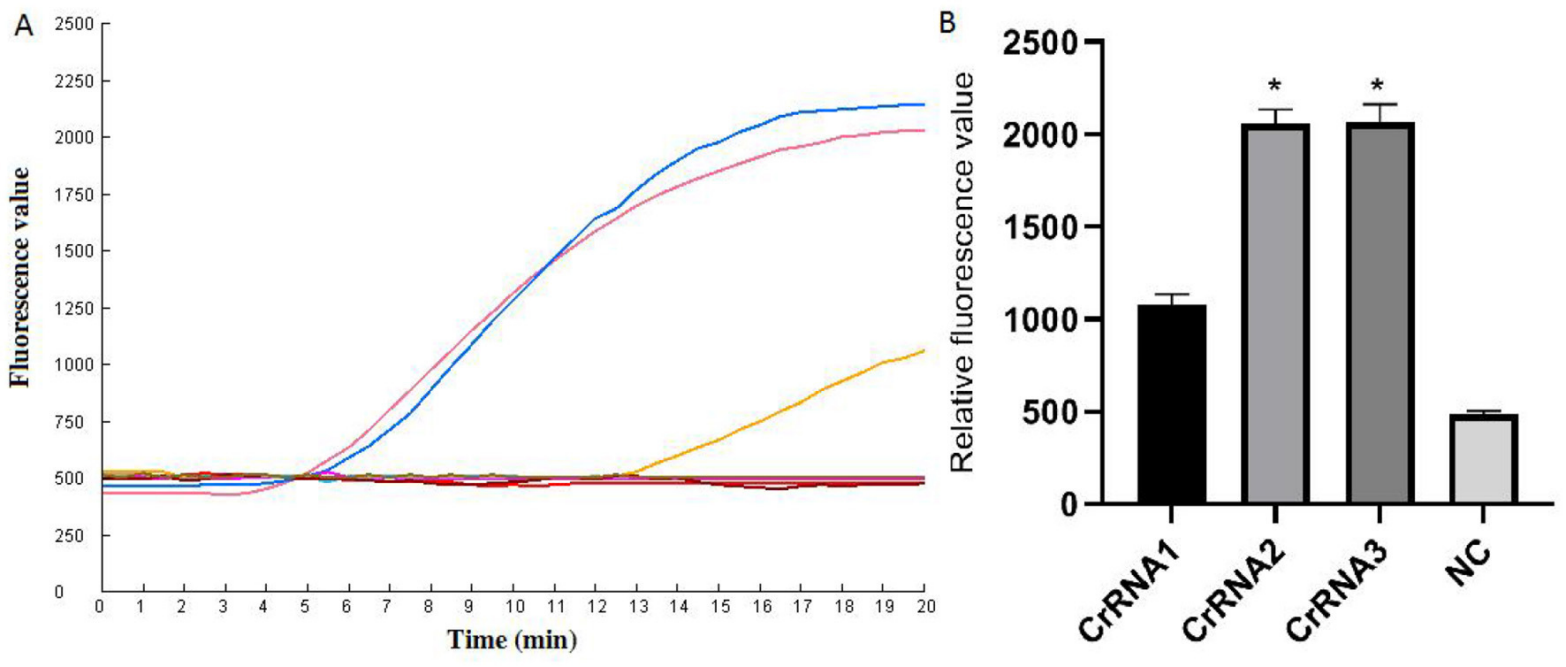
Screening of the optimal CrRNA. **(A)** Evaluation of the effect of the CrRNAs by the Cas12a detection assay. **(B)** The tests were conducted in triplicate. ^*^Represents a *p*-value less than 0.05.

The screening of CrRNA will utilize the constructed plasmid pMD-Hexon as a template for detection. The assay was conducted separately using the three CrRNAs (CrRNA1, CrRNA2 and CrRNA3). The fluorescence levels of the reaction products were assessed. As illustrated in Figure 3, results demonstrate that all three CrRNAs generate fluorescence curves. Notably, the fluorescence values for CrRNA2 and CrRNA3 surpassed that of CrRNA1. Meanwhile, the fluorescence values for CrRNA2 and CrRNA3 were the same as each other. Consequently, CrRNA2 was selected for subsequent experiments. The alignment of the CrRNA2 sequence was illustrated in Figure 2A.

### Reaction conditions

**Figure 4:**
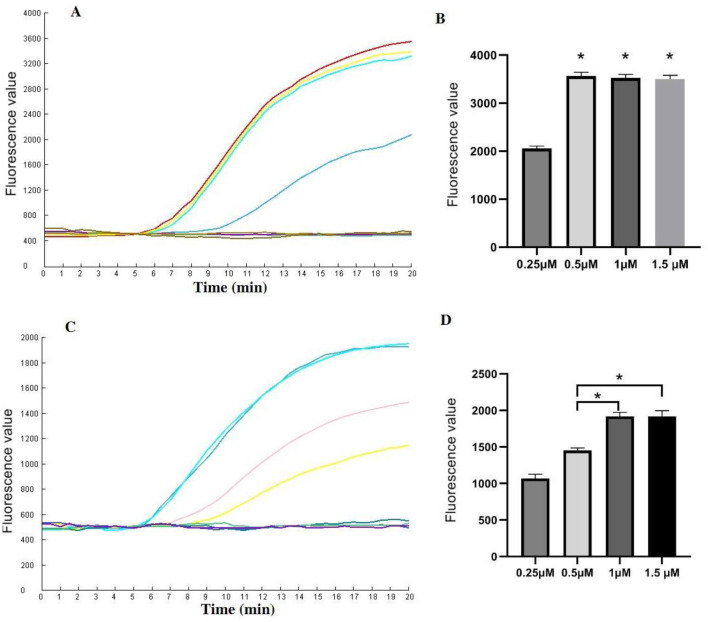
Evaluation of the CrRNA and Cas12a concentrations in the one-tube RPA-CRISPR/Cas12a test. **(A, B)** Different CrRNA concentrations were tested at 0.25 μM, 0.5 μM, 1 μM and 1.5 μM. The tests were conducted in triplicate; **(C, D)** Cas12a concentrations were evaluated at 0.25 μM, 0.5 μM, 1 μM and 1.5 μM. ^*^Represents a *p*-value less than 0.05.

Various concentrations of CrRNA and Cas12a were assessed. The results demonstrated that fluorescence intensity increased with increasing concentrations of Cas12a (1 μM, 0.5 μM, 0.25 μM). However, no significant differences in peak fluorescence intensity were observed among the higher concentrations of Cas12a (2 μM, 1.5 μM, 1 μM). Therefore, a Cas12a concentration of 1 μM was selected for subsequent assays (Figures 4A, B).

Next, the optimal concentration of CrRNA was investigated. As illustrated in Figures 4C, D, the use of higher concentrations of CrRNA (1 μM, 0.5 μM, 0.25 μM) accelerated the time required to achieve exponential amplification. When the CrRNA concentration exceeded 1 μM, there were no significant differences in the time to achieve exponential amplification. Consequently, a CrRNA concentration of 1 μM was chosen for the follow-up experiments.

### Sensitivity

**Figure 5:**
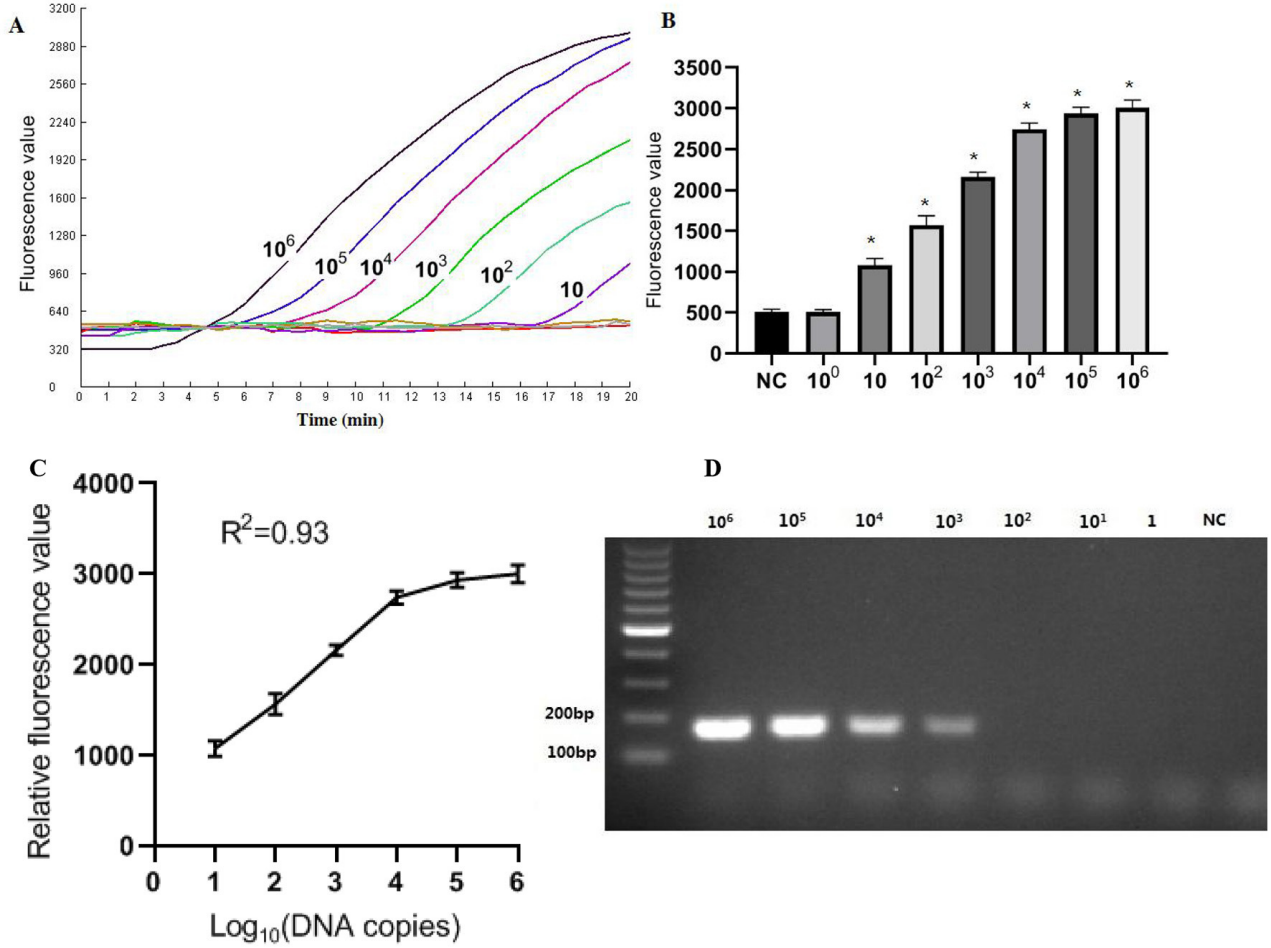
Sensitivity results based on fluorescence detection. **(A)** To determine the minimum detection of the RPA-CRISPR/Cas12a assay, ten-fold dilution standards were detected as templates by the method. **(B)** The tests were conducted in triplicate. **(C)** The LoD of the basic RPA assay. **(D)** Semi-logarithmic regression of the data collected from 3 runs using the DNA standards was analyzed by GraphPad Prism 5.0. ^*^Represents a *p*-value less than 0.05.

The recombinant plasmid pMD-Hexon was serially diluted and used as a template for sensitivity detection in the RPA-CRISPR/Cas12a method, while the established PCR method served as a comparison. As illustrated in Figure 5, the real-time RPA-CRISPR/Cas12a method can detect as low as 10 copies/μL. When utilizing template concentrations ranging from 10 to 10^6^ copies, the method exhibits distinct exponential amplification curves. The fluorescence signal is detectable within a time frame of 5–15 min, indicating the method's rapid responsiveness in identifying target pathogen across this concentration range. The sensitivity of the detection method developed in this study is comparable to that of the qPCR method. However, when using template concentrations of 10 to 10^6^ copies in qPCR, the time range for fluorescence amplification signals to appear is around 20–35 min. The data collected from three runs with the DNA standards were analyzed using semi-logarithmic regression in GraphPad Prism 5.0. The analysis demonstrated that this assay is reproducible (Figure 5C). However, the limit of detection (LoD) for the basic RPA was 10^3^ copies/μL, which is 100 times lower than that of the one-tube RPA-CRISPR/Cas12a method (Figure 5D).

### Repetitive testing

The results indicated that the intra-group coefficients of variation for the different concentrations of molecular standards were 3.0%, 2.7%, and 7.5%, while the inter-group coefficients of variation were 1.9%, 4.0%, and 7.0% (Table 2). Both the intra-group and inter-group coefficients of variation (CV) were below 10%, demonstrating that the method established in this experiment exhibits good reproducibility.

**Table 2:** Repeatability test.

**DNA copies**	**Inter-repeat test**		**Intra-repeat test**	
	**Fluorescence average value ±SD**	**CV**	**Fluorescence average value ±SD**	**CV**
10^6^	3,005 ± 96	3.0%	3,032 ± 57	1.90%
10^4^	2,744 ± 73	2.7%	2,768 ± 110	4.00%
10^2^	1,567 ± 116	7.5%	1,594 ± 110	7.00%

### Specificity test

**Figure 6:**
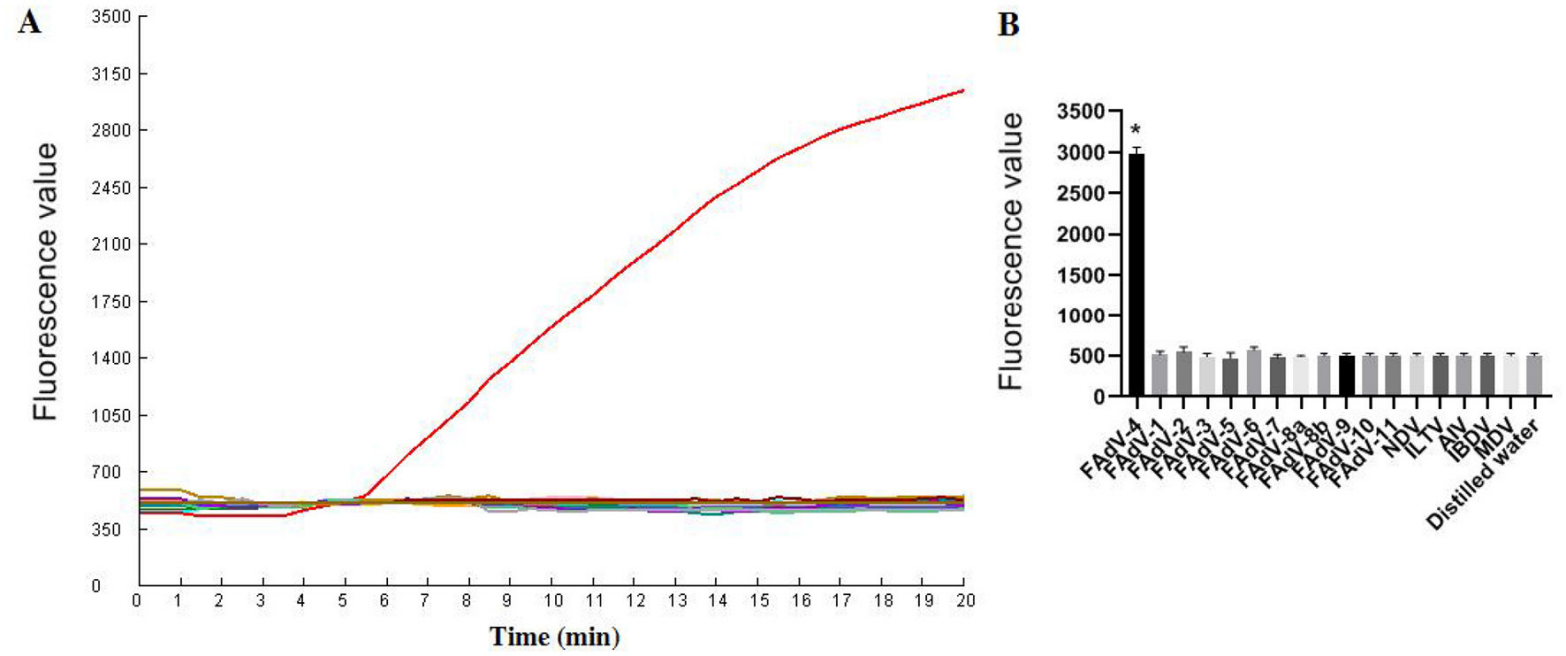
Specificity results based on fluorescence detection. **(A)** FAdV-4 and other common avain viruses (AIV, NDV, ILTV, MDV, IBDV, FAdV-1, FAdV-2, FAdV-3, FAdV-5, FAdV-6, FAdV-7, FAdV-8a, FAdV-8b, FAdV-9, FAdV-10, FAdV-11) were detected by the RPA CRISPR/Cas12a assay; **(B)** The tests were conducted in triplicate. ^*^Represents a *p*-value less than 0.05.

As illustrated in Figure 6, only FAdV-4 tested positive by the one-tube RPA-CRISPR/Cas12a method. All the other viruses (AIV, NDV, ILTV, MDV, IBDV, FAdV-1, FAdV-2, FAdV-3, FAdV-5, FAdV-6, FAdV-7, FAdV-8a, FAdV-8b, FAdV-9, FAdV-10, FAdV-11) tested negative by the assay. These results demonstrate that the one-tube RPA-CRISPR/Cas12a method exhibited high specificity, as only the target virus produced positive result, with no cross-reactivity observed with other common pathogens.

### Clinical sample testing results

Upon completion of the DNA extraction, the RPA-CRISPR/Cas12a assay was performed to assess the presence of FAdV-4 in the clinical samples. The results indicated that 18 out of the 40 samples tested positive for FAdV-4, demonstrating a high prevalence of the virus among the affected chickens. Among these 18 positive samples, 9 heart samples and 9 liver samples from 9 chickens were positive. This result indicates that the method has the same effect on detecting different samples. The samples were also analyzed using qPCR, revealing that out of the 18 samples that tested positive for RPA, real-time qPCR confirmed 17 samples as positive, with CT values ranging from 21 to 31; one sample returned a negative result. Conversely, among the 22 samples that tested negative via RPA, qPCR also identified them all as negative. The agreement rate between the qPCR assay and the RPA assay for clinical samples was 97.5% (Table 3).

**Table 3:** Comparison of the detection results between RPA-CRISPR/Cas12a and qPCR.

		**qPCR**		**Total**	**CR**
		**Positive**	**Negative**		
RPA-CRISPR/ Cas12a	Positive	17	1	18	
	Negative	0	22	22	97.5%
	Total	17	23	40	

CR, coincidence rate. CR = (17+22)/40.

## Discussion

FAdV-4 is a highly contagious virus affecting various bird species, especially poultry and wild birds, causing avian inclusion body hepatitis (IBH) and hepatitis-hydropericardium syndrome (HHS). The development of rapid diagnostic tools for detecting FAdV-4 is essential for effective disease control and eradication efforts. In this study, we have developed a RPA assay in combination with CRISPR/Cas12a, specifically targeting the Hexon gene. The RPA-CRISPR/Cas12a assay can detect as few as 10 genomic copies per reaction, demonstrating high sensitivity comparable to qPCR. Additionally, the assay displays high specificity for FAdV-4, with no cross-reactivity observed with other common avian pathogens.

Numerous studies have explored a range of methods for detecting FAdV-4. PCR-RFLP involves cutting the amplified PCR product with specific endonucleases and then visualizing the results using gel electrophoresis (Raue and Hess, 1998; Mase et al., 2010). Raue et al. designed two pairs of primers targeting the conserved region of the Hexon gene in 1998. This was followed by amplification of the gene and digestion with the Hae II enzyme, allowing for the differentiation of 12 FAdV reference strains (Raue and Hess, 1998). Mase et al. (2010) also designed primers targeting the fiber-1 gene of FAdV-4 and FAdV-10. They utilized PCR-RFLP combined with the Alu I enzyme to differentiate between FAdV-4 and FAdV-10. However, these methods of amplification followed by digestion with endonucleases were relatively complex and may not be suitable for processing a large number of clinical samples.

Mase et al. (2021) established a routine gel-based PCR detection method enabling the simultaneous identification of FAdV-1, FAdV-2, FAdV-4, FAdV-8a, and FAdV-8b. Wang et al. (2017a) designed two primers and a probe based on the FAdV-4 Hexon gene sequence to establish a qPCR detection method, achieving a minimum detection limit of 10 copies/μL. Zhai et al. (2019) designed two pairs of primers based on the sequence of conserved region of the 52K gene and developed a LAMP assay for the detection of FAdV-4, with a detection limit of 10 copies/μL.

Despite the establishment of these detection methods, they are primarily suited for laboratory settings and are not ideal for regions with inadequate infrastructure and limited resources. This limitation poses significant challenges for effectively preventing and controlling FAdV-4 infection. To address these issues, this study developed a rapid detection method for FAdV-4 using RPA in combination with CRISPR/Cas12a, thus compensating for the aforementioned shortcomings.

Literature reviews have demonstrated that CRISPR/Cas12a has become a widely utilized and emerging diagnostic tool in veterinary clinical testing. Typically, before conducting CRISPR/Cas12a testing, the target gene is first amplified using nucleic acid amplification methods (Huang et al., 2023; Kostyusheva et al., 2022; Yin et al., 2024).

Conventional PCR and isothermal amplification methods, including RPA and LAMP, are effective techniques for amplifying target genes for DNA detection. However, PCR requires multiple thermal cycles in a thermal cycler for amplification. LAMP generally operates within a consistent temperature range of 60–66°C; however, its complex primer design and susceptibility to non-specific effects can hinder its suitability for on-site applications. In contrast, RPA allows for the simultaneous opening of DNA double strands and synthesis of complementary strands, enabling amplification to be completed in just 20 min, making it particularly well-suited for on-site deployment (Ma et al., 2020, 2023; Zhu et al., 2022; Ma et al., 2022; Tan et al., 2022; Li et al., 2018; Wang J. C. et al., 2017; Wang et al., 2017c,b). While RPA does carry a risk of non-specific amplification, the addition of CrRNA significantly enhances its specificity (Kellner et al., 2019).

The diagnostic method established in the present study operates within a single reaction tube. Initially, RPA is employed to amplify the target fragment, which can be incubated at 39°C for 20 min, with no special instrument requirements during the reaction process. Subsequently, The CRISPR/Cas12a components, positioned on the tube lid, were driven into the tube by centrifugal force, resulting in a cleavage reaction of the ssDNA reporter molecule. This detection occurs within a single reaction tube, allowing for analysis with a fluorescence detector without the need to open the tube. This approach not only simplifies the procedure but also minimizes the risk of potential aerosol contamination. Previous studies typically employed a two-step approach, separating RPA amplification from CRISPR/Cas12a detection (Liu et al., 2021; Zhao et al., 2023; Talwar et al., 2021). The two-step method can be cumbersome and susceptible to various influencing factors, potentially resulting in biased outcomes. In contrast, the one-tube method used in this study streamlines the process, reduces operational complexity, minimizes the risk of error, enhances detection efficiency, and supports broader application in clinical settings.

A RPA assay for detecting FAdV has been reported (Zhang et al., 2020). The amplification process was completed in just 14 min, marking a significant improvement over conventional PCR, which typically requires 98 min. However, similar to PCR, the RPA assay still necessitates agarose gel electrophoresis for result analysis, which takes ~30 min to prepare and run. Consequently, when comparing the gel-based RPA method to conventional PCR, there are no substantial advancements in operational convenience. In contrast, our study established a real-time assay that eliminates the need for electrophoresis following amplification, significantly enhancing efficiency. A previous study described a detection method that combines RPA with test strips for virus identification. While this approach is effective, it necessitates opening the reaction tube and transferring the RPA product to the test strip for detection. This process not only extends the overall experimental time but also heightens the risk of aerosol contamination (Jirawannaporn et al., 2022).

This new detection method for FAdV-4 is designed for on-site testing. Our study integrates RPA and CRISPR/Cas12a technology to develop a novel diagnostic method that offers an alternative assay for the early monitoring of FAdV-4 and the epidemiological investigation of local chicken populations. The real-time RPA-CRISPR/Cas12a assay is user-friendly, highly specific, and sensitive, requiring no specialized instruments or equipment. Its accessibility makes it particularly suitable for implementation in remote areas. Therefore, this research establishes a simple, efficient, rapid, and accurate method for detecting FAdV-4, representing a significant advancement in the prevention and control of this virus.

## Data Availability

The original contributions presented in the study are included in the article/supplementary material, further inquiries can be directed to the corresponding author.
